# Force Field Analysis Software and Tools (FFAST): Assessing Machine Learning Force Fields under the Microscope

**DOI:** 10.1021/acs.jctc.3c00985

**Published:** 2023-11-27

**Authors:** Gregory Fonseca, Igor Poltavsky, Alexandre Tkatchenko

**Affiliations:** Department of Physics and Materials Science, University of Luxembourg, Luxembourg City L-1511, Luxembourg

## Abstract

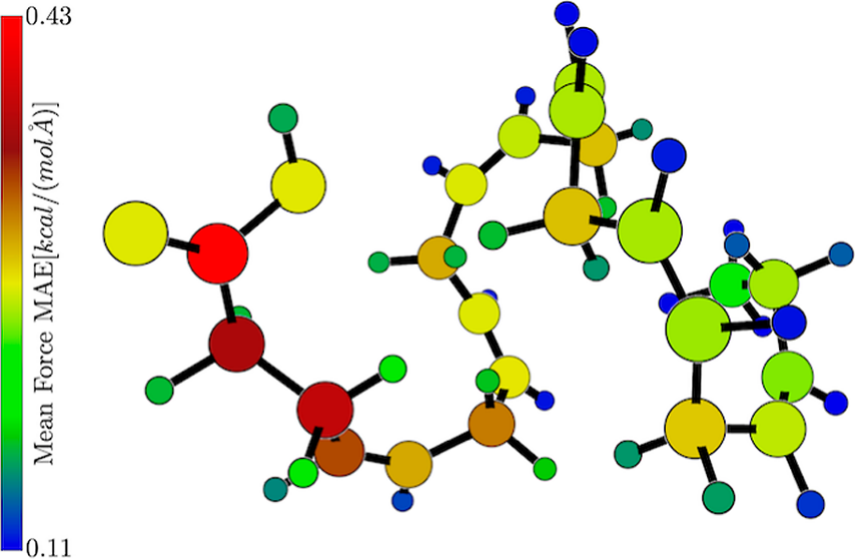

As the sophistication of machine learning force fields (MLFF) increases to match the complexity of extended molecules and materials, so does the need for tools to properly analyze and assess the practical performance of MLFFs. To go beyond average error metrics and into a complete picture of a model’s applicability and limitations, we developed FFAST (force field analysis software and tools): a cross-platform software package designed to gain detailed insights into a model’s performance and limitations, complete with an easy-to-use graphical user interface. The software allows the user to gauge the performance of any molecular force field,—such as popular state-of-the-art MLFF models, — on various popular data set types, providing general prediction error overviews, outlier detection mechanisms, atom-projected errors, and more. It has a 3D visualizer to find and picture problematic configurations, atoms, or clusters in a large data set. In this paper, the example of the MACE and NequIP models is used on two data sets of interest [stachyose and docosahexaenoic acid (DHA)]—to illustrate the use cases of the software. With this, it was found that carbons and oxygens involved in or near glycosidic bonds inside the stachyose molecule present increased prediction errors. In addition, prediction errors on DHA rise as the molecule folds, especially for the carboxylic group at the edge of the molecule. We emphasize the need for a systematic assessment of MLFF models for ensuring their successful application to the study of dynamics of molecules and materials.

## Introduction

Many of the cornerstones of modern advancements in industry and technology rely on the introduction of novel or optimized materials, newly designed drugs, and the understanding of physicochemical phenomena on a molecular level. Here, computer simulations and predictions of properties play a pivotal role in enabling, enhancing, or accelerating research and development.^1−11^

Amidst the computational tools, machine learning force fields (MLFF) are steadily rising in popularity, with a wide variety of models achieving remarkable predictive accuracy^12−52^ for various molecules and materials of ever-increasing sizes and complexity.^53−59^ The ultimate goal of MLFF is to bring the quality of expensive ab initio methods to systems of larger scales, which is currently mostly feasible for efficient but significantly less accurate empirical mechanistic force fields. MLFFs aim to advance FF development by learning highly accurate data generated by state-of-the-art quantum chemistry methods to then reproduce their results in a fraction of the computational time. However, in order to use these models in practice, it is crucial to understand for which tasks a given MLFF is appropriate. ML models are very sensitive to their training data, the training procedure, hyperparameters, and many other details: two models with similar overall accuracy but trained on different data, hyperparameters, or architectures can still present fundamental differences in actual applications (for example, molecular dynamics) that are not captured by simple error metrics.^60,61^

For practical applications, it is important to know when our model is accurate and when it is likely to fail. This information is crucial given that a single configuration that the model predicts inaccurately can move the system under study into an improbable or unphysical state, which could affect the remaining simulation. The probability of such a misprediction is steadily growing with increasing size and chemical and structural complexity of the systems under study. While no silver bullets exist to make ML models perfectly stable, analyzing models beyond usual error metrics can avoid many pitfalls and limitations. This is why we developed FFAST (force field analysis software and tools): a cross-platform software package designed to gain detailed insights into a model’s performance and limitations, complete with an easy-to-use graphical user interface. Note that while this paper’s main focus is MLFFs, all the described tools are equally applicable to empirical force fields or, in fact, force fields of any kind.

The review is organized as follows: in the Software section, the software and its components are listed and briefly explained. In the Workflow section, part of those components is explored in a realistic use-case from start to finish. The Applications section applies the above workflow to actual data sets and models and analyzes the results. Conclusions and outlooks can be found in the final section.

## Software

Out of the box, FFAST can load a variety of MLFF models (currently supported sGDML,^29^ SchNet,^20^ NequIP,^12,13^ MACE,^14,15^ SpookyNet,^27^ and prepredicted forces/energies), as well as data set formats (currently supporting.xyz, .npz, and .db). The ML models are used to generate predictions of the energy and forces on the loaded data sets. To prevent excessive loading times when handling large data sets or expensive models, this step can be accomplished in headless mode (i.e., without a graphical interface) to precompute the predictions externally (e.g., on a high-performance computer).

Once loaded, various analysis tools are available in a user-friendly interface.

- Error distributions on both energy and force predictions are available, able to visualize multiple data set/model combinations for easy comparisons. By default, mean average errors (MAE) are used, and the plots visualize a Gaussian kernel-density estimate.
- Error timelines show the MAE across the given time-ordered data set (e.g., a dynamic). An adjustable smoothing factor is provided to disregard fluctuations by averaging each point over the given number of neighbors.
- Plots of cluster errors provide a way to find problematic regions of configurational space. By default, agglomerative clustering on pairwise interatomic distances is followed up by KMeans on energies, for a total of 40 clusters.
- Error scatter plots are available to find outliers quickly, both for energies and forces.
- Distribution of atomic errors can be used to determine the difference in prediction between chosen elements. The procedure is otherwise identical to error distributions.

The user can zoom in on most plots and choose to create subdata sets. These are subsets of loaded data sets to be further analyzed by the software. Subsets and data sets can be saved in any format that is provided by the software in case external editing or analysis is needed.

The program comes equipped with a 3D visualization tool. The molecular structures inside of a data set or subset can be viewed in one or more interactive windows. The visualizer comes with convenience features like aligning geometries along a chosen plane for easier visualization, choosing the bonds to be visualized, or animating the molecule throughout the selected data set. One can extract information on the geometry such as atomic distances, angles or dihedrals. The user can enable the plotting of force prediction errors on each atom of the molecule, either at the given time frame or averaged throughout the data set/subset. Finally, atoms of interest can be selected and analyzed independently in all of the analysis tools above.

One can also use the above-described tools to analyze the underlying reference data set by creating a dummy model. In this case, FFAST will operate on the reference energy and forces instead of the difference between the reference data and ML models’ prediction.

The software is designed to be modular, allowing users comfortable with Python to add features that they need for their workflow. The workflow is streamlined such that swapping between software codes and separate scripts for different models is eliminated as much as possible. The only dependencies are those of the external ML models themselves (subject to their own installation process) and readily available Python packages. As such, FFAST provides a platform that beginners and experts alike can use to quickly and precisely assess their force field performance in an unbiased way.

## Workflow

To illustrate the usefulness of the program, an example workflow is presented in this chapter. It is important to note that this use-case serves to guide and highlight important points of the software, not to be an all-inclusive exposition of all its features.

As a first step, one or multiple ML models and data sets of interest can be loaded. If the data sets are large or the MLFFs are expensive, the program can be used in headless mode on a supercomputer to precompute the forces and energies. This allows the user to avoid unnecessary recalculations and prolonged loading times while using the software. For all applications in this paper, data for molecular systems are taken from the MD22 data set.^58^ These data sets contain the set of all atomic coordinates as well as the atomic forces and total potential energies at every step of a computed trajectory. This section specifically focuses on its stachyose trajectory as an example. The molecule (C_24_H_42_O_21_) consist of 87 atoms of carbon, oxygen, or hydrogen and appears as one of the most common tetrasaccharides in plants and is such an important sugar. The particular selection of models used as illustrations in this paper is NequIP and MACE. The parameters used for all models reflect those recommended in the original paper or the official software description of the respective packages. In all cases, all models are trained on the same 1000 training points for each data set, respectively.

### Prediction Error Overview

**Figure 1:**
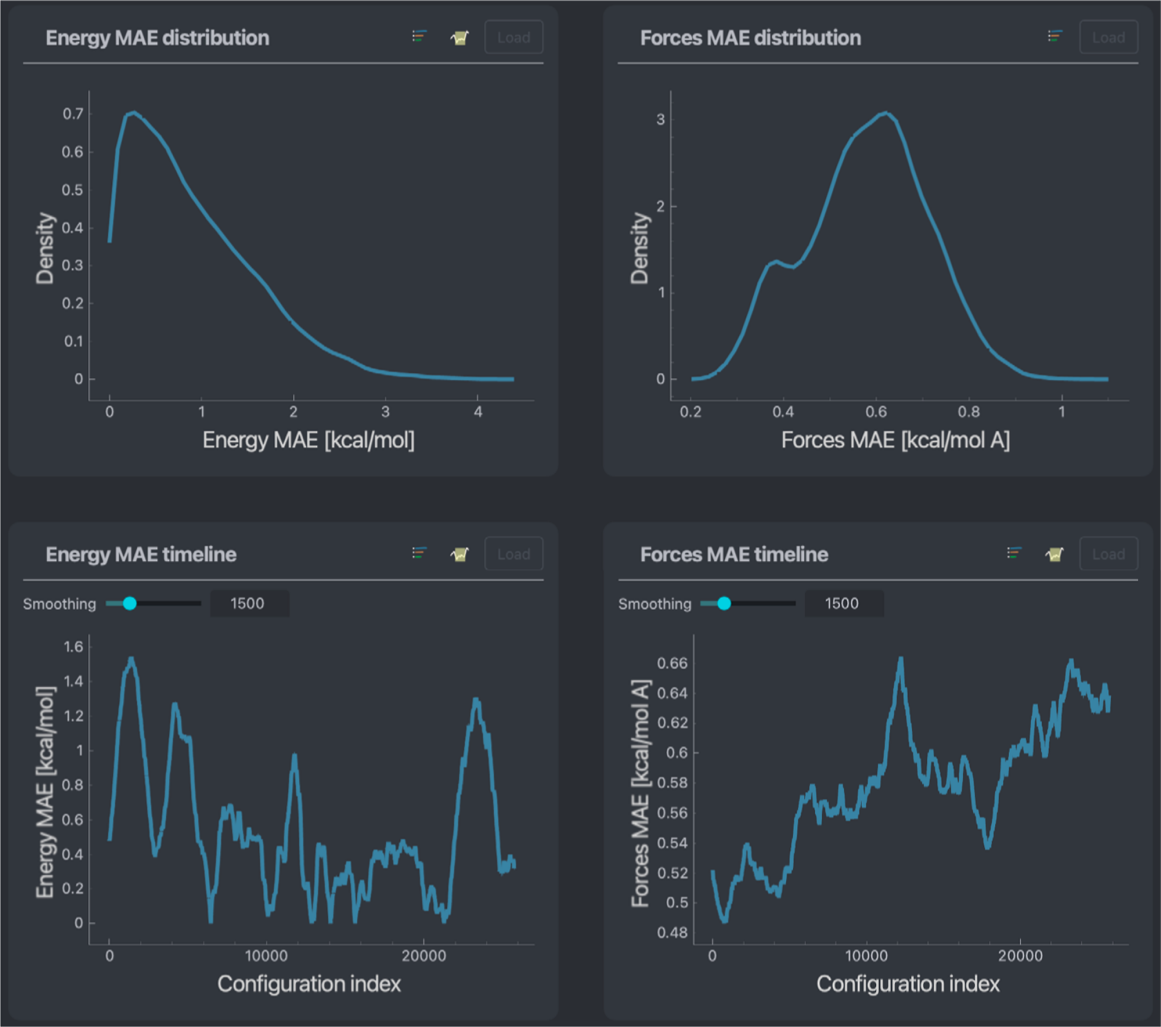
Basic error screen including timelines and distributions for both energy and forces, MAEs across an entire stachyose data set. In blue, a NequIP model trained on 1000 points of the data set is used.

The first screen to greet the user contains basic information, providing an overview of a model’s overall performance. This overview includes basic error metrics such as MAEs, root mean squared errors (RMSEs), error distributions, and error timelines throughout a data set. An example is given in Figure 1. Error distributions are one of the fastest ways to determine a model’s performance. From a glance, they provide a rough estimate of the accuracy across a given data set and what to expect for the low and high end of the error spectrum. Furthermore, one can look at the distribution patterns (such as deviation from a normal curve) to understand whether a systematic error occurs on specific data subsets.

Error timelines show the prediction errors throughout the data set’s lifecycle. This is mostly useful if the data are generated from molecular dynamic simulations, as it allows the user to observe general trends or rough moments in time where an MLFF model’s performance might have been lacking. The average window size can be adjusted manually to find a good balance between coarseness and precision.

### Outlier Detection

Many tools are provided to find outliers in the data set or in the prediction error profile. While average errors across entire data sets are helpful, they do not provide any indication as to why and where the FF fails. As such, it is essential to determine which configurations cause issues and whether those are outliers in the data set or important representative geometries.

Correlation scatters are scatter plots of the predicted values against true values. While simple, they allow users to visually determine which points, likely far from the expected linear correlation, are worth a second look. Besides, the general shape of a correlation plot gives a good indication of the stability of the model, as in practice, a single bad prediction during a time step can result in the entire dynamics steering into the nonphysical territory.

**Figure 2:**
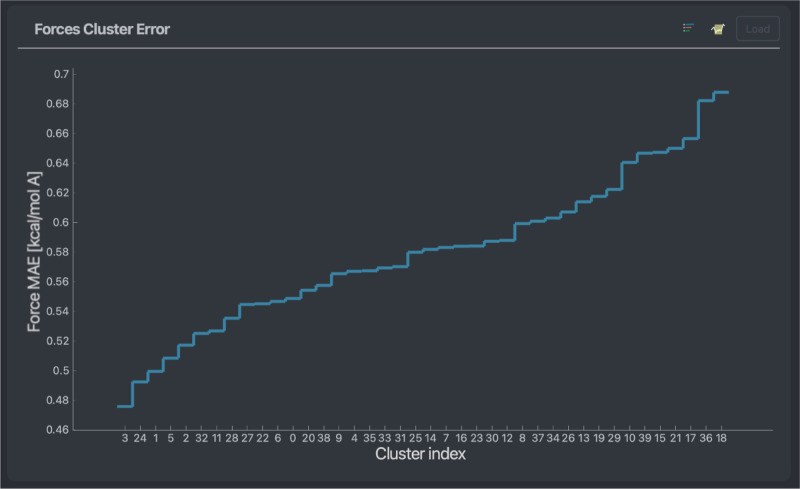
Mean average error of force predictions of a NequIP model trained on 1000 points for 50 different clusters (ordered by ascending error) of a stachyose data set.

A more in-depth way to visualize a model’s stability across areas of configurational space available in a given data set is through the usage of clustering algorithms,^60^ see the example in Figure 2. In essence, the data set is split into a select number of distinct clusters, each containing a varying number of configurations. The groups are chosen such as to maximize the similarity between configurations of the same group, thereby splitting the entire data sets into a digestible number of qualitatively different configurations. The total number of clusters is a parameter that can be adjusted in FFAST. For the systems in this work, 40 clusters were empirically found to be a good compromise leading to clusters small enough to be qualitatively distinct but large enough to be meaningfully represented in the data set. Error analysis of those different clusters provides an overview akin to an error distribution; however, its discrete nature focuses on types of configuration rather than single points. Thus, the user can apply the chemical intuition and knowledge of the molecule at hand to potentially find a) why a model fails on certain clusters but not others, b) what the application range of the model is, or c) outliers in the data set.

### Atomic Errors

**Figure 3:**
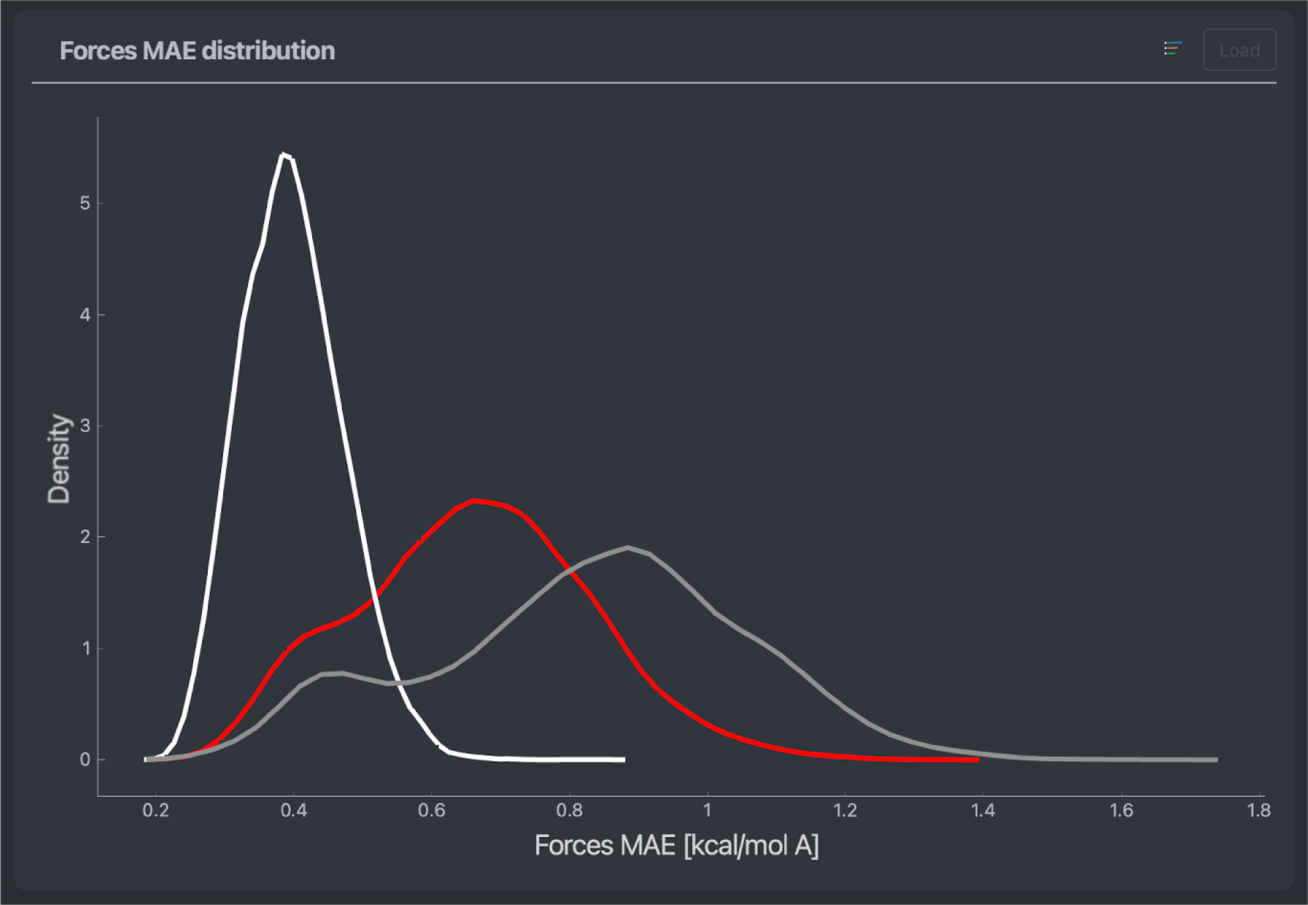
Mean average error density of the force predictions of a NequIP model trained on 1000 points of a stachyose data set. The densities are measured separately for each atom type. Colors correspond to elements (Hydrogen: white, Oxygen: red, Carbon: gray).

While the previous plots focus on finding single or groups of configurations with particular prediction error patterns, there is also merit to focusing on models’ performance for different atom types. With the increasing chemical composition and structural complexity of the systems of interest for MLFFs, atoms of the same type interact differently with their environment based on their environment composition. Thus, one can expect that the ML model predictions vary across atom types. FFAST provides the advantage of visualizing a given model’s force prediction error distribution across different chemical elements of the system. Alternatively, one can view the error distribution for a selected chemical element across multiple ML models or data sets. An example can be found in Figure 3.

### 3D Visualization

All the previous features offered a way to get a general understanding of FF performance and find outliers for the models. However, to extract chemical intuition from these results, it is paramount to visualize them in a 3D space. FFAST allows most of its plots mentioned above to be used for visualization purposes. Zoom into any subarea of interest and visualize only configurations within that region. Beyond giving form to the points, areas, and clusters of interest, the 3D visualization tool allows for configuration-by-configuration and atom-by-atom basis analysis. One can view the force prediction error for each atom on an individual geometry or errors averaged throughout all selected configurations. That way, if a specific mechanism is at the root of poor predictions, combining outlier detection (to find the geometries involved) and atomic force prediction error (to see the atoms involved) can provide a path to revealing its cause.

## Applications

After demonstrating the main features of the FFAST, we will apply it to analyze in detail the capabilities of two state-of-the-art ML architectures, namely, NequIP and MACE, to reconstruct PESs and FFs of flexible organic molecules. While both ML models can easily reach below chemical-accuracy MAEs on precomputed test data sets, obtaining stable long-time dynamics is a nontrivial task. Therefore, understanding the origin of prediction errors on an atomistic level is crucial for accurate application and model prediction improvements.

### Stachyose

This subsection focuses on the stachyose molecule, and the primary ML architecture is the NequIP model trained on the aforementioned data set and analyzed using the FFAST software. The basic errors screen, (see Figure 1) reveals some noteworthy trends. Most of the configurations in the data set fall within chemical accuracy for the chosen model, as indicated by the energy MAE distribution’s main contributor lying below 1 kcal/mol. This also applies to the force MAE distribution with the overall MAE of 0.58 kcal/(mol Å). Interestingly, the force error distribution demonstrates a well-defined double-peak shape, suggesting nonequal force reconstruction for different system components. Also, the forces and energy MAE timelines show oscillating behavior clearly distinguishing sets of configurations, reflecting that the molecular dynamics that generated the data set explored qualitatively different molecular structures throughout the simulation instead of oscillating in or around an equilibrium state.

The atomic error plot in Figure 3 expands on the overall distributions and distinctly shows a fundamental difference between hydrogen and other elements. The overall MAE and RMSE values of each atom type can be found in Table 1. Importantly, the well-predicted hydrogen atoms, making up almost half of the molecule, highly influence the overall prediction errors. This also largely explains the double-peak structure found in the overall force error distribution discussed above. Nevertheless, the secondary peaks in the distributions are also visible on a per-atom basis, notably for carbon but also in oxygen. Note that the inhomogeneities in prediction errors between atom types (and even within atom types) can partly be explained by the varying average force amplitudes that the respective atoms feel. Nevertheless, an analysis of the relative errors, i.e., normalized by the average force vector norm for each respective atom type, reveals that the inhomogeneous prediction errors are not fully explained by the scales of the force amplitudes.

**Table 1:** Overall Force MAE and RMSE in kcal/(mol) for Each Atom Type in the Stachyose Data set, as Predicted by a NequIP Model of 1000 Training Points^a^

	H	C	C_b_	C_r_	C_s_	O	O_b_	O_r_	O_s_	All
MAE	0.40	0.83	0.92	0.78	0.77	0.66	0.89	0.82	0.57	0.58
RMSE	0.55	1.10	1.23	1.04	1.02	0.90	1.20	1.10	0.76	0.82

a The subscripts b, r, and s indicate filtering for only atoms touching a glycosidic bond, inside the rest of the ring, or in a side chain, respectively.

**Figure 4:**
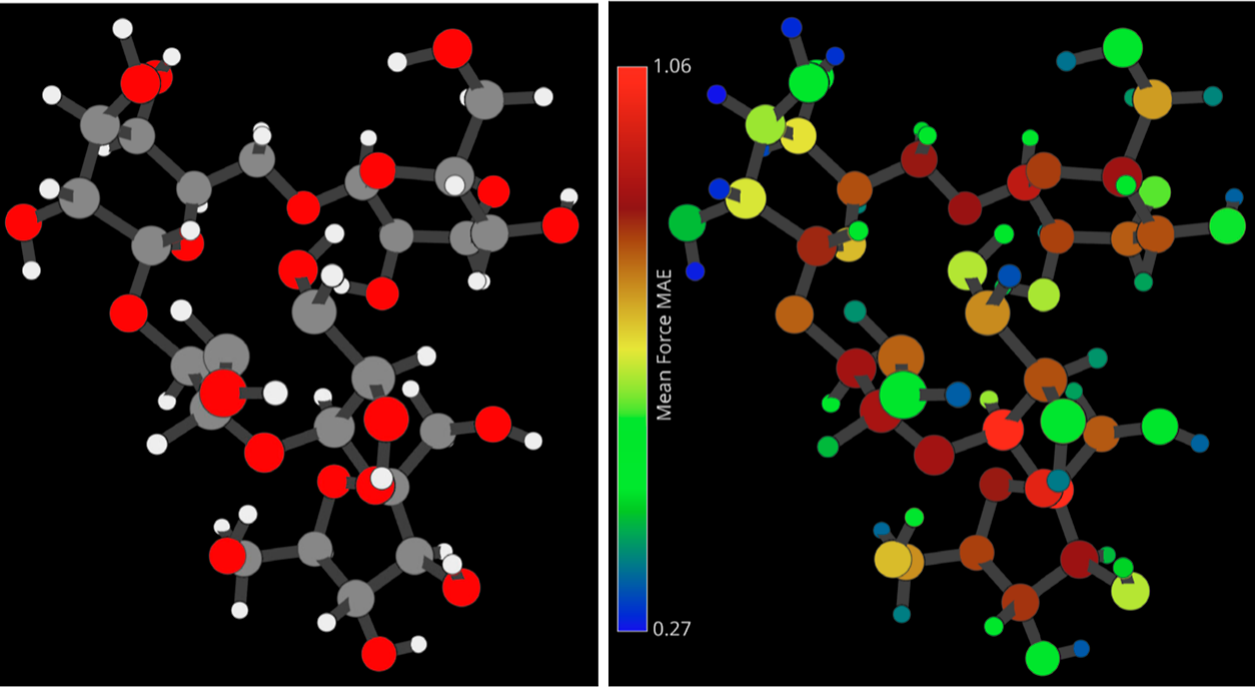
Cutout of the 3D visualizer on the stachyose molecule. (Left) atoms colored according to their element. (Right) atoms colored according to their respective mean average force error as predicted by a NequIP model trained on 1000 points.

The reason for the double-peak structure of carbon and oxygen force error distributions is revealed when looking at the average force prediction error for every individual atom in the 3D visualizer; see Figure 4 (right). The molecule consists of a main chain of one furanose (5-membered carbohydrate ring with oxygen) and three pyranoses (6-membered carbohydrate ring with oxygen). Upon inspection, one can notice that the carbons involved in or directly touching the glycosidic bonds between the rings have notably worse errors than those elsewhere on the rings (by about ∼18%). A similar situation is found for the oxygens inside said bonds, which have worse predictions than those in the rings or, more significantly, than those in side-chain hydroxyl groups (by about ∼56%, see Table 1). Remembering that a model’s actual simulation performance is largely defined by its most problematic atoms rather than an overall mean error is important. Hence, it would be wise to consider that in practical applications such as molecular dynamics, the global structure of the molecule would primarily be determined by the atoms in the main chain, whose force MAE and RMSE are ∼0.9 and ∼1.2 kcal/(mol Å) respectively. Also, based on this analysis, one could consider introducing atom types based on their chemical environment rather than purely by its nuclear charge, akin to methods employed for empirical force fields with chemically diverse systems.^62^

**Figure 5:**
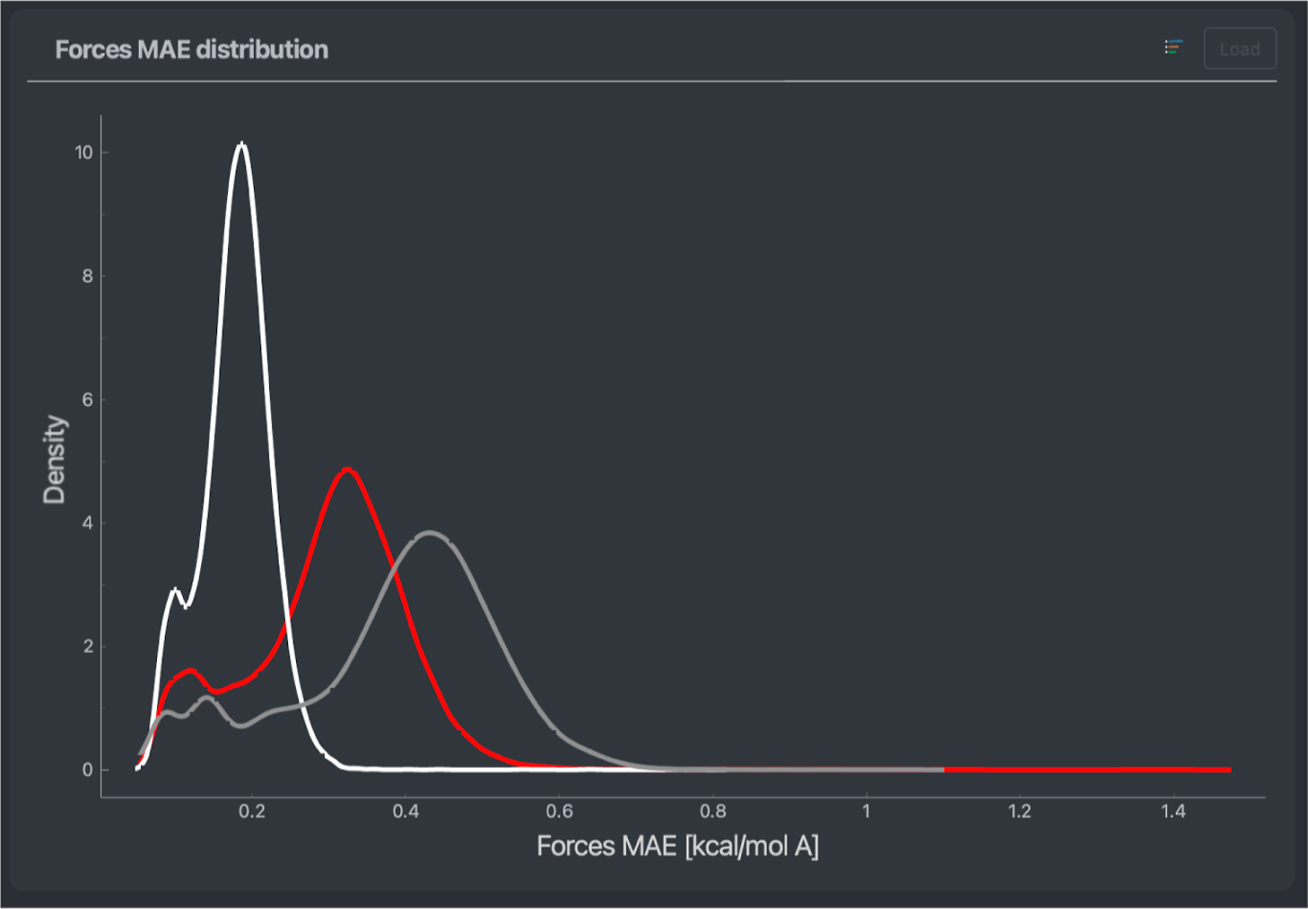
Mean average error density of the force predictions of a MACE model trained on 1000 points of a stachyose data set. The densities are done separately for each atom type. Colors correspond to elements (Hydrogen: white, Oxygen: red, Carbon: gray).

All of the prior discussions can be repeated with a separate model, e.g., a MACE model, created with the same training set. In Figure 5, one can see that a very similar trend can be observed, with even more distinguishable peaks in the error distributions. Also, all the observations made in the 3D visualizer for the MACE model are qualitatively identical to those made for the NequIP model, showing significant similarity in both models’ performance for the considered system.

### Docosahexaenoic Acid

**Figure 6:**
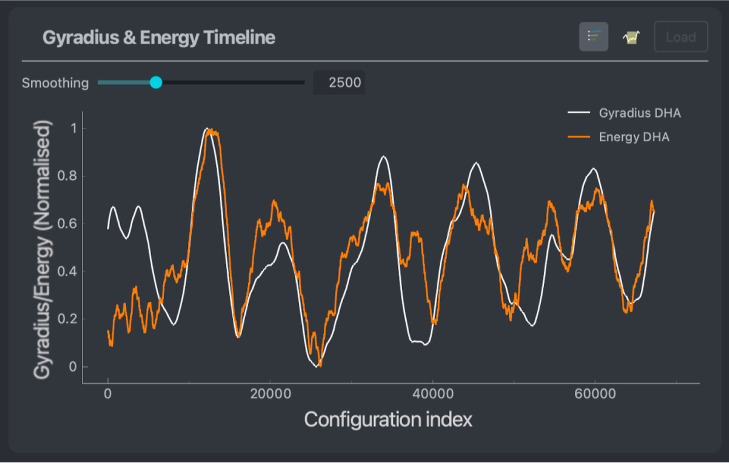
Gyradius (white) and energy (orange) throughout the trajectory of a DHA molecule. Values are averaged over a window of 2500 points to smooth out noise.

A second example illustrating the use-case of the FFAST is that of the docosahexaenoic acid (DHA) molecule, a fatty acid with the chemical formula C_22_H_32_O_2_ for a total of 56 atoms. The molecule comprises a 22-carbon chain with six double bonds and a carboxylic head. DHA is rather flexible due to its long hydrocarbon tail, meaning that the molecule can visit various extended and folded states at ambient conditions. Therefore, understanding how well the folding and unfolding processes are represented in the reference data set is crucial for constructing a reliable MLFF. FFAST provides a simple tool to visualize the folding/unfolding processes by tracking the molecule’s radius of gyration (or gyradius), as shown in Figure 6. This does not require any ML model to be loaded. Figure 6 shows that, in total, the data set consists of six compact and six extended states. Moreover, the radius of gyration correlates with the molecule’s potential energy after an equilibration period of approximately 10,000 simulation steps. One region where the correlation is broken is the fourth folded state (around 40k step). While the molecule is compact, its potential energy is relatively high for those configurations, suggesting the occurrence of some nontrivial interaction patterns demanding further investigation. Interestingly, both ML models trained in this subsection demonstrate the largest energy MAEs and noticeable force MAEs strictly for this set of structures, fortifying the previous conclusion.

**Figure 7:**
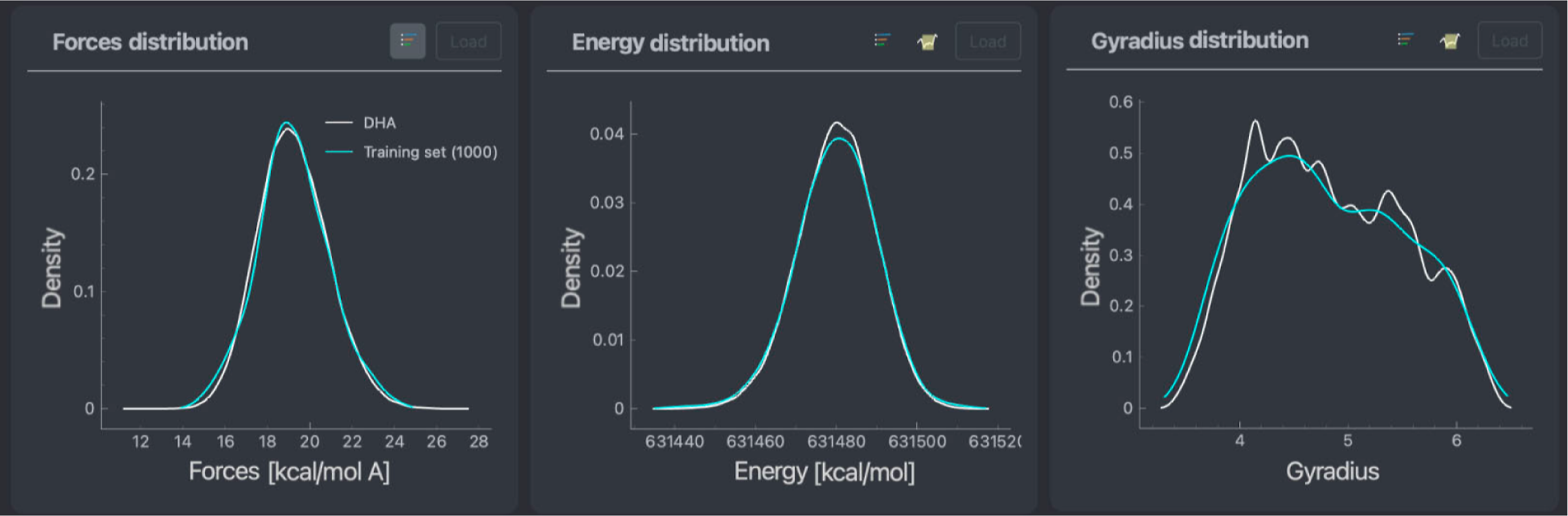
Force distribution (left), energy distribution (middle), and gyradius distribution (right) of the entire DHA data set (white) against a training set of 1000 points (cyan). The training set was generated using the sGDML training point selection method.

Another essential analysis that can quickly be performed using FFAST and is a prerequisite for training reliable MLFF for flexible molecules: ensuring the quality of the training set. Here, we compare the distributions of gyration radius, forces, and energies within the given (complete) data set and the generated training (sub)set of 1000 points; see Figure 7. The comparison verifies that the training set is indeed representative of the data set and the obtained ML model should not unexpectedly enter interpolation regimes while within the reference’s configurational space.

**Figure 8:**
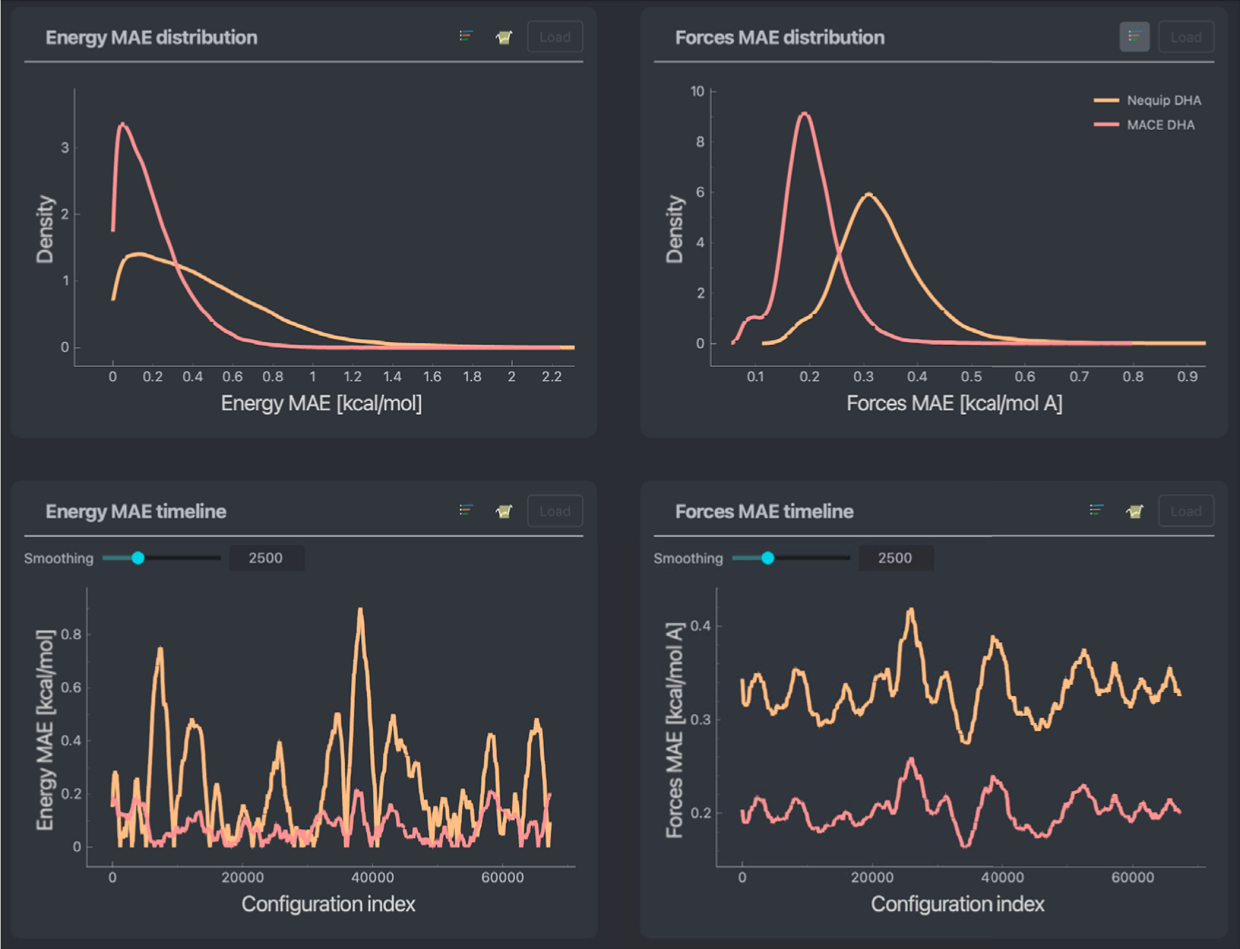
Basic error screen including timelines and distributions for both energy and forces MAEs across an entire stachyose data set. In orange and red, respectively, NequIP and MACE models trained on 1000 points of the data set are used.

With such an insight into the content of the given reference and training data sets, we can build MLFFs aiming at the description of folded and unfolded DHA geometries and the transitions between them. Below, we compare the performance of two competing state-of-the-art ML architectures under the same conditions. In Figure 8, one can see NequIP (orange) and MACE (red), both generated using the same training and validation set of 1000 points each. One can observe that in this case, the MACE model has better general accuracy than the NequIP equivalent, with each presenting an overall MAE on forces of 0.20 and 0.33 kcal/(mol Å), respectively. Furthermore, the error distribution peaks for the MACE model are narrower for both the energy and forces. Interestingly, the difference in accuracy does not come from a better prediction of forces acting on a specific type of atom within the MACE model. A similar 50% decrease in the MAEs is observed for hydrogens, oxygens, and carbons, as can be seen by comparing atomic errors (similar to the analyses shown in Figure 3). This hints at the overall better performance of the MACE architecture for reconstructing the PES of the DHA molecule.

**Figure 9:**
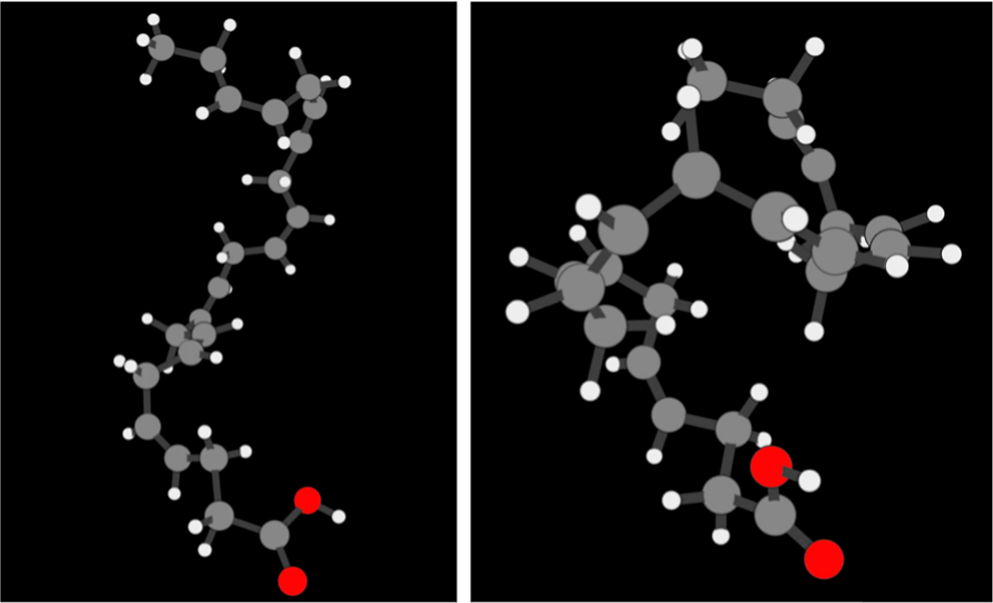
(Left) Example of an extended DHA molecule, as found in the valleys of the MAE timeline and low-error clusters. (Right) Example of a folded DHA molecule, as found in the peaks of the MAE timeline and high-error clusters. The geometries were chosen from the lowest and highest force prediction error cluster, respectively.

Notably, distinct loosely periodic peaks can be observed in both the energy and force MAE timeline. These peaks match between NequIP and MACE, meaning that they are unlikely to be due to an artifact of the models’ predictions but rather a fundamental change in geometry throughout those time steps. Zooming in on the valleys/peaks and displaying the configurations in the 3D visualizer reveal that the low error regions correspond to extended geometries while the high error peaks contain folded geometries. An example for each can be seen in Figure 9.

**Figure 10:**
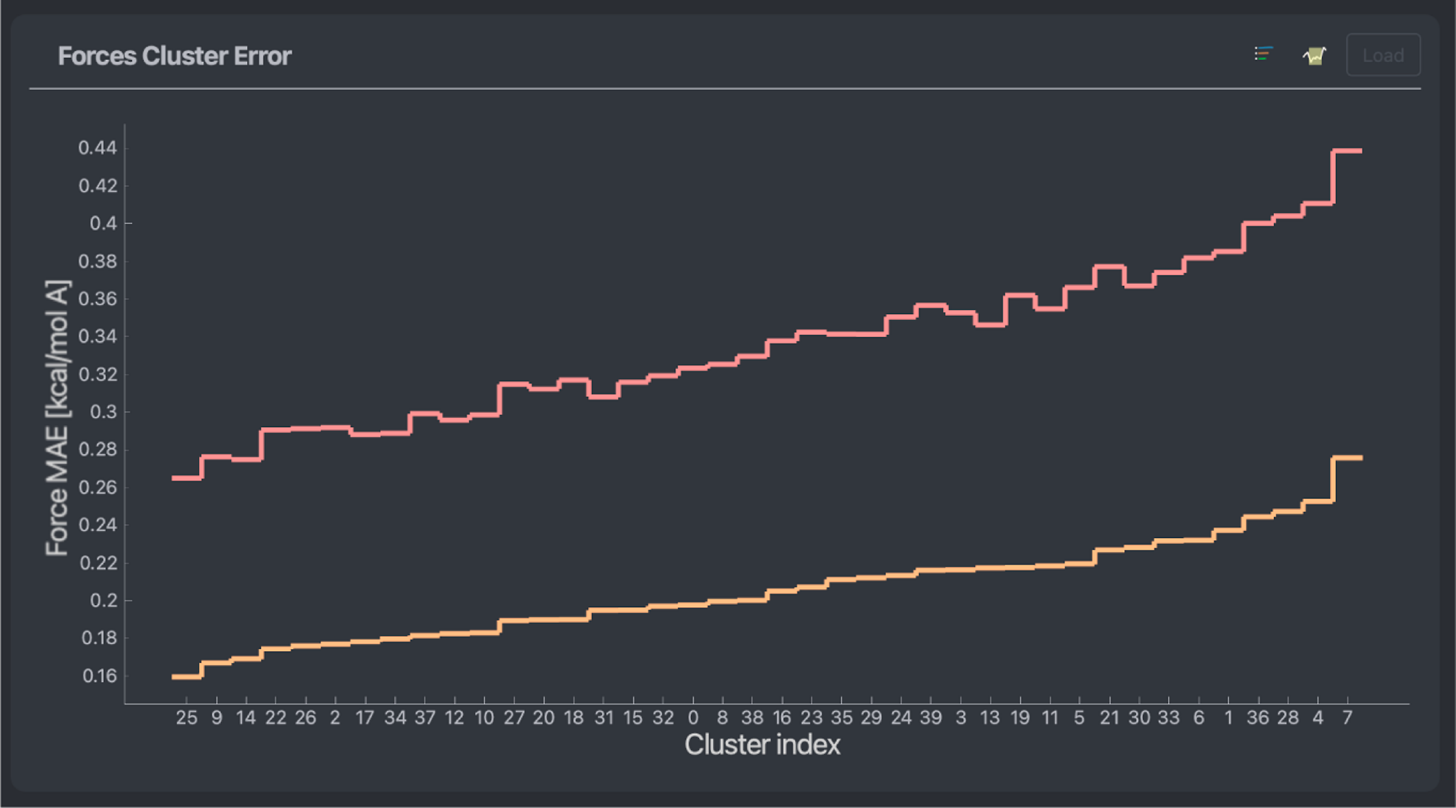
Mean average error of force predictions for 50 different clusters of a stachyose data set. The models used are a NequIP model (red) and a MACE model (orange) trained on 1000 points. The clusters are ordered in ascending prediction error of the MACE model.

The mean force prediction cluster errors (see Figure 10) show a relatively flat profile; i.e., the highest average force prediction error on a group of similar geometries is less than two times higher than that of the lowest. Nevertheless, similarly to the error timeline plots, visualizing the high error clusters consistently displays folded configurations and vice versa. One can find an example of the lowest and highest error clusters can be found in Figure 9.

**Figure 11:**
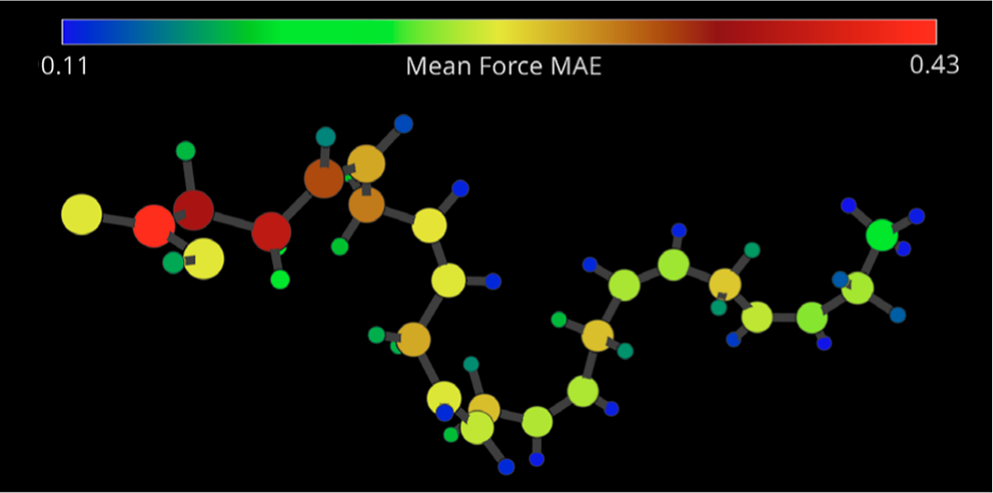
Extended configuration of a DHA molecule. Atoms are colored according to their respective mean average force error, as predicted by a MACE model.

Finally, additional useful information can be obtained by visualizing the average force prediction error on every atom in the 3D visualizer, as shown in Figure 11. Once again, hydrogens are very well predicted across the board, suggesting that a significant contribution to the low overall prediction error comes from the 32 hydrogen atoms in the molecule. In contrast, one can see that the carbon atoms inside a carboxylic group and those closest to it (on the left side of the figure) are significantly worse predicted than the rest of the chain. While not shown here, the NequIP model presents a similar overall trend. One can easily explain the observed nonuniform force reconstruction for carbon atoms (both for NequIP and MACE) by combining the information obtained from the FFAST software and our physical/ML intuition. The carbon atoms near the head of the DHA molecule containing oxygen atoms are exposed to a more complex chemical composition of their neighborhood than those in other parts of the molecule. Thus, the descriptor space for such carbon atoms contains more possible states, especially in folded-like configurations. This leads to noticeably more challenging training tasks for the ML models resulting in larger prediction errors. This again hints at the need to reconsider the definition of atomic types based on the atom’s environment composition rather than its nuclear weight when we move from simple periodic systems or small molecules to more challenging systems.

All in all, our analysis shows that beyond the insignificant for most practical applications error difference in less than 0.25 kcal/mol for the energy and 0.20 kcal/(mol) for the forces, both the NequIP and MACE models demonstrate equivalent results for the given test problem. Therefore, one can in principle apply any of these two models to study molecules similar to DHA or stachyose examples.

## Outlook

The applications in this paper (as well as many other examples omitted) demonstrate that increased chemical and structural complexity of mid- and large-size molecules or periodic systems require a detailed analysis of MLFFs’ stability, reliability, and performance. For such cases, standard metrics such as the MAE and RMSE are only one piece of the puzzle. High overall accuracies for reconstructing a PES or FF can largely stem from the good performance of an ML model on specific (albeit large) parts of the system; see the case of hydrogens in the examples above. At the same time, forces for other vital system components might be predicted with a significantly lower accuracy. For both examples of DHA and stachyose, the largest errors for carbon atoms lie in a region where the overall force error distribution shows negligible probability. This effect is clearly observed despite the molecules containing less than a hundred atoms. For more complex systems, such as molecules on surfaces, periodic structures containing rigid crystal frames, molecular interstitials, etc., the difference between mean errors and region-specific errors could become even more prominent. When looking to enhance models and go beyond current capabilities, all of these effects can heavily influence the next directions to undertake. To create a reliable way to systematically improve ML models, one first needs to agree exactly what the current limitations are and which areas need targeted improvements. Furthermore, reaching certain limitations does not necessarily mean that the employed ML architectures cannot handle the given tasks. With a detailed insight into the models’ performance, one can often significantly increase its applicability by adjusting the training process or the underlying data. Therefore, FFAST and similar software packages should become a staple tool for ML model developers and users.

Another challenge where FFAST can be indispensable is assessing the stability of the MLFFs MD trajectories. Providing stable long-term dynamics for complex systems is not at all guaranteed, even for state-of-the-art MLFFs. A long enough simulation eventually reaches a region where extrapolation is necessary, which is likely to generate unphysical forces and heavily influence the rest of the dynamics. These effects can sometimes be easily detected (e.g., breaking of bonds, dissociated hydrogen atoms, etc.), but less obvious consequences can also follow. Namely, unphysical behavior can occur without showing apparent damage to the molecular structure during the MD run. Physical/chemical properties computed thereafter would be affected without clear evidence to the user that extrapolation regions were reached. While some techniques, such as predicting the model uncertainty, can partially alleviate this problem, the fact that the given ML model is confident in its prediction does not necessarily mean that the prediction is correct. The outlier detection tools and interactive visualization implemented in FFAST provide an efficient alternative instrument to detect such situations without significant human effort. Considering that MLFFs by construction focus first on the most typical interaction patterns, such analyses also highlight nontrivial physics and chemistry that might be present in the system. Therefore, combining FFAST with modern MLFF architectures should also enable a better understanding of the interplay between different types of interactions in large and complex systems.

## Conclusions

With the increased complexities of systems that can be simulated using novel MLFFs comes the need to provide insightful analysis for these models. Even the most advanced MLFFs demonstrate highly heterogeneous predictions across the configurational space (CS), invisible to overall error metrics. Thus, detailed breakdowns of the ML models’ performance on different domains of CS and various parts of the system under study should become standard practice. FFAST is an actively developing tool allowing experts and nonexperts to get an in-depth insight into the performance of MLFFs or any other force field of choice on any data set. In this paper, a list of provided features is given and explained, including the example of a best practice scenario. The features were showcased using two data sets of medium-sized flexible organic molecules: docosahexaenoic acid and stachyose.

Two state-of-the-art ML models (NequIP and MACE) were trained for each data set. With those, a general FFAST workflow was showcased, and the following conclusions followed. Hydrogens are generally significantly better predicted for both molecules than for other atoms, considerably lowering the overall ML models’ MAEs and RMSEs in hydrogen-rich systems. In contrast, forces acting on carbons and oxygens are unevenly reconstructed. Atoms with chemically or structurally more diverse environments present significantly higher prediction errors compared with the other atoms of the same type. For instance, the prediction errors on DHA rise as the molecule folds, and the primary source of the errors comes from the carboxylic group.

Beyond a comprehensive analysis of the stability and reliability of ML models, FFAST provides a means to analyze reference data. For instance, one can easily find the number of folding/unfolding processes in a given MD trajectory and the correlation of that with the potential energy. Another possible application is comparing energy/force distributions in different data sets (training, validation, and test), which should precede any ML model training. As FFAST offers detailed reference data sets and MLFF error analysis, field-specialized knowledge can be applied to enhance MLFFs after identifying the remaining challenges. The software is provided as an open-source project on github.com/fonsecag/FFAST and is designed to be modulable and expandable to adapt to the user’s needs.

## References

[ref1] Lee A. C.; Harris J. L.; Khanna K. K.; Hong J.-H. A Comprehensive Review on Current Advances in Peptide Drug Development and Design. Int. J. Mol. Sci. 2019, 20, 2383 10.3390/ijms20102383. 31091705 PMC6566176

[ref2] Ramos J.; Muthukumaran J.; Freire F.; Paquete-Ferreira J.; Otrelo-Cardoso A. R.; Svergun D.; Panjkovich A.; Santos-Silva T. Shedding Light on the Interaction of Human Anti-Apoptotic Bcl-2 Protein with Ligands through Biophysical and in Silico Studies. Int. J. Mol. Sci. 2019, 20, 860 10.3390/ijms20040860. 30781512 PMC6413030

[ref3] Mansbach R. A.; Travers T.; McMahon B. H.; Fair J. M.; Gnanakaran S. Snails In Silico: A Review of Computational Studies on the Conopeptides. Mar. Drugs 2019, 17, 145 10.3390/md17030145. 30832207 PMC6471681

[ref4] Jing Z.; Liu C.; Cheng S. Y.; Qi R.; Walker B. D.; Piquemal J.-P.; Ren P. Polarizable Force Fields for Biomolecular Simulations: Recent Advances and Applications. Annu. Rev. Biophys. 2019, 48, 371–394. 10.1146/annurev-biophys-070317-033349. 30916997 PMC6520134

[ref5] Ekins S. The Next Era: Deep Learning in Pharmaceutical Research. Pharm. Res. 2016, 33, 2594–2603. 10.1007/s11095-016-2029-7. 27599991 PMC5042864

[ref6] Smalley E. AI-powered Drug Discovery Captures Pharma Interest. Nat. Biotechnol. 2017, 35, 604–605. 10.1038/nbt0717-604. 28700560

[ref7] Elton D. C.; Boukouvalas Z.; Butrico M. S.; Fuge M. D.; Chung P. W. Applying Machine Learning Techniques to Predict the Properties of Energetic Materials. Sci. Rep. 2018, 8, 9059 10.1038/s41598-018-27344-x. 29899464 PMC5998124

[ref8] Elton D. C.; Boukouvalas Z.; Fuge M. D.; Chung P. W. Deep Learning for Molecular Design—a Review of the State of the Art. Mol. Syst. Des. Eng. 2019, 4 (4), 828–849. 10.1039/C9ME00039A.

[ref9] Faber F. A.; Lindmaa A.; von Lilienfeld O. A.; Armiento R. Machine Learning Energies of 2 Million Elpasolite $(AB{C}_{2}{D }_{6})$ Crystals. Phys. Rev. Lett. 2016, 117, 135502 10.1103/PhysRevLett.117.135502. 27715098

[ref10] Naserifar S.; Chen Y.; Kwon S.; Xiao H.; Goddard W. A. III Artificial Intelligence and QM/MM with a Polarizable Reactive Force Field for Next-Generation Electrocatalysts. Matter 2021, 4, 195–216. 10.1016/j.matt.2020.11.010.

[ref11] Cova T. F. G. G.; Pais A. A. C. C. Deep Learning for Deep Chemistry: Optimizing the Prediction of Chemical Patterns. Front. Chem. 2019, 7, 809 10.3389/fchem.2019.00809. 32039134 PMC6988795

[ref12] Batzner S.; Musaelian A.; Sun L.; Geiger M.; Mailoa J. P.; Kornbluth M.; Molinari N.; Smidt T. E.; Kozinsky B. E. E(3)-equivariant graph neural networks for data-efficient and accurate interatomic potentials. Nat. Commun. 2022, 13, 2453 10.1038/s41467-022-29939-5. 35508450 PMC9068614

[ref13] Geiger M.; Smidt T. E3nn: Euclidean Neural Networks. 2022, arXiv:2207.09453.

[ref14] Batatia I.; Kovacs D. P.; Simm G. N. C.; Ortner C.; Csanyi G. MACE: Higher Order Equivariant Message Passing Neural Networks for Fast and Accurate Force Fields; NeurIPS, 2022.

[ref15] Batatia I.; Batzner S.; Kovács D. P.; Musaelian A.; Simm G. N. C.; Drautz R.; Ortner C.; Kozinsky B.; Csányi G. The Design Space of E(3)-Equivariant Atom-Centered Interatomic Potentials. 2022, arXiv:2205.06643. 10.1038/s42256-024-00956-x PMC11769842 39877429

[ref16] Bartók A. P.; Payne M. C.; Kondor R.; Csányi G. Gaussian Approximation Potentials: The Accuracy of Quantum Mechanics, without the Electrons. Phys. Rev. Lett. 2010, 104, 136403 10.1103/PhysRevLett.104.136403. 20481899

[ref17] Bartók A. P.; Kondor R.; Csányi G. On Representing Chemical Environments. Phys. Rev. B 2013, 87, 184115 10.1103/PhysRevB.87.184115.

[ref18] Behler J. Perspective: Machine Learning Potentials for Atomistic Simulations. J. Chem. Phys. 2016, 145, 170901 10.1063/1.4966192. 27825224

[ref19] Behler J.; Parrinello M. Generalized Neural-Network Representation of High-Dimensional Potential-Energy Surfaces. Phys. Rev. Lett. 2007, 98, 146401 10.1103/PhysRevLett.98.146401. 17501293

[ref20] Schütt K. T.; Kindermans P.-J.; Sauceda H. E.; Chmiela S.; Tkatchenko A.; Müller K.-R. SchNet: A Continuous-Filter Convolutional Neural Network for Modeling Quantum Interactions. 2017, arXiv:1706.08566.

[ref21] Schütt K. T.; Arbabzadah F.; Chmiela S.; Müller K. R.; Tkatchenko A. Quantum-Chemical Insights from Deep Tensor Neural Networks. Nat. Commun. 2017, 8, 13890 10.1038/ncomms13890. 28067221 PMC5228054

[ref22] Schütt K. T.; Sauceda H. E.; Kindermans P.-J.; Tkatchenko A.; Müller K. R. SchNet – A Deep Learning Architecture for Molecules and Materials. J. Chem. Phys. 2018, 148, 241722 10.1063/1.5019779. 29960322

[ref23] Schütt K. T.; Kessel P.; Gastegger M.; Nicoli K. A.; Tkatchenko A.; Müller K. R. SchNetPack: A Deep Learning Toolbox For Atomistic Systems. J. Chem. Theory Comput. 2019, 15, 448–455. 10.1021/acs.jctc.8b00908. 30481453

[ref24] Rupp M.; Tkatchenko A.; Müller K. R.; von Lilienfeld O. A. Fast and Accurate Modeling of Molecular Atomization Energies with Machine Learning. Phys. Rev. Lett. 2012, 108, 058301 10.1103/PhysRevLett.108.058301. 22400967

[ref25] Bartók A. P.; De S.; Poelking C.; Bernstein N.; Kermode J. R.; Csányi G.; Ceriotti M. Machine Learning Unifies the Modeling of Materials and Molecules. Sci. Adv. 2017, 3, 1701816 10.1126/sciadv.1701816. PMC5729016 29242828

[ref26] Unke O. T.; Meuwly M. PhysNet: A Neural Network for Predicting Energies, Forces, Dipole Moments, and Partial Charges. J. Chem. Theory Comput. 2019, 15, 3678–3693. 10.1021/acs.jctc.9b00181. 31042390

[ref27] Unke O. T.; Chmiela S.; Gastegger M.; Schütt K. T.; Sauceda H. E.; Müller K. R. SpookyNet: Learning Force Fields with Electronic Degrees of Freedom and Nonlocal Effects. Nat. Commun. 2021, 12, 7273 10.1038/s41467-021-27504-0. 34907176 PMC8671403

[ref28] Unke O. T.; Chmiela S.; Sauceda H. E.; Gastegger M.; Poltavsky I.; Schütt K. T.; Tkatchenko A.; Müller K. R. Machine Learning Force Fields. Chem. Rev. 2021, 121, 10142–10186. 10.1021/acs.chemrev.0c01111. 33705118 PMC8391964

[ref29] Chmiela S.; Sauceda H. E.; Müller K. R.; Tkatchenko A. Towards Exact Molecular Dynamics Simulations with Machine-Learned Force Fields. Nat. Commun. 2018, 9, 3887 10.1038/s41467-018-06169-2. 30250077 PMC6155327

[ref30] Chmiela S.; Sauceda H. E.; Poltavsky I.; Müller K. R.; Tkatchenko A. sGDML: Constructing Accurate and Data Efficient Molecular Force Fields Using Machine Learning. Comput. Phys. Commun. 2019, 240, 38–45. 10.1016/j.cpc.2019.02.007.

[ref31] Sauceda H. E.; Gálvez-González L. E.; Chmiela S.; Paz-Borbón L. O.; Müller K. R.; Tkatchenko A. BIGDML—Towards Accurate Quantum Machine Learning Force Fields for Materials. Nat. Commun. 2022, 13, 3733 10.1038/s41467-022-31093-x. 35768400 PMC9243122

[ref32] Qiao Z.; Welborn M.; Anandkumar A.; Manby F. R.; Miller T. F. OrbNet Deep Learning for Quantum Chemistry Using Symmetry-Adapted Atomic-Orbital Features. J. Chem. Phys. 2020, 153, 124111 10.1063/5.0021955. 33003742

[ref33] Smith J. S.; Isayev O.; Roitberg A. E. ANI-1: An Extensible Neural Network Potential with DFT Accuracy at Force Field Computational Cost. Chem. Sci. 2017, 8, 3192–3203. 10.1039/C6SC05720A. 28507695 PMC5414547

[ref34] Devereux C.; Smith J. S.; Huddleston K. K.; Barros K.; Zubatyuk R.; Isayev O.; Roitberg A. E. Extending the Applicability of the ANI Deep Learning Molecular Potential to Sulfur and Halogens. J. Chem. Theory Comput. 2020, 16, 4192–4202. 10.1021/acs.jctc.0c00121. 32543858

[ref35] Deringer V. L.; Bernstein N.; Bartók A. P.; Cliffe M. J.; Kerber R. N.; Marbella L. E.; Grey C. P.; Elliott S. R.; Csányi G. Realistic Atomistic Structure of Amorphous Silicon from Machine-Learning-Driven Molecular Dynamics. J. Phys. Chem. Lett. 2018, 9, 2879–2885. 10.1021/acs.jpclett.8b00902. 29754489

[ref36] Ryczko K.; Mills K.; Luchak I.; Homenick C.; Tamblyn I. Convolutional Neural Networks for Atomistic Systems. Comput. Mater. Sci. 2018, 149, 134–142. 10.1016/j.commatsci.2018.03.005.

[ref37] Frank J. T.; Unke O. T.; Müller K.-R. So3krates: Equivariant Attention for Interactions on Arbitrary Length-Scales in Molecular Systems. 2023, arXiv:2205.14276.

[ref38] Christensen A. S.; Bratholm L. A.; Faber F. A.; Anatole von Lilienfeld O. FCHL Revisited: Faster and More Accurate Quantum Machine Learning. J. Chem. Phys. 2020, 152, 044107 10.1063/1.5126701. 32007071

[ref39] Wang J.; Olsson S.; Wehmeyer C.; Pérez A.; Charron N. E.; de Fabritiis G.; Noé F.; Clementi C. Machine Learning of Coarse-Grained Molecular Dynamics Force Fields. ACS Cent. Sci. 2019, 5 (5), 755–767. 10.1021/acscentsci.8b00913. 31139712 PMC6535777

[ref40] Ko T. W.; Finkler J. A.; Goedecker S.; Behler J. General-Purpose Machine Learning Potentials Capturing Nonlocal Charge Transfer. Acc. Chem. Res. 2021, 54, 808–817. 10.1021/acs.accounts.0c00689. 33513012

[ref41] Kocer E.; Ko T. W.; Behler J. Neural Network Potentials: A Concise Overview of Methods. Annu. Rev. Phys. Chem. 2022, 73, 163–186. 10.1146/annurev-physchem-082720-034254. 34982580

[ref42] Gasteiger J.; Groß J.; Günnemann S. Directional Message Passing for Molecular Graphs. 2022, arXiv:2003.03123. arXiv preprint.

[ref43] Gasteiger J.; Becker F.; Günnemann S. GemNet: Universal Directional Graph Neural Networks for Molecules. 2022, arXiv:2106.08903.

[ref44] Deringer V. L.; Bartók A. P.; Bernstein N.; Wilkins D. M.; Ceriotti M.; Csányi G. Gaussian Process Regression for Materials and Molecules. Chem. Rev. 2021, 121, 10073–10141. 10.1021/acs.chemrev.1c00022. 34398616 PMC8391963

[ref45] Bartók A. P.; Kermode J.; Bernstein N.; Csányi G. Machine Learning a General-Purpose Interatomic Potential for Silicon. Phys. Rev. X 2018, 8, 041048 10.1103/physrevx.8.041048.

[ref46] Thomas N.; Smidt T.; Kearnes S.; Yang L.; Li L.; Kohlhoff K.; Riley P. Tensor Field Networks: Rotation- and Translation-Equivariant Neural Networks for 3D Point Clouds. 2018.

[ref47] Khorshidi A.; Peterson A. A. Amp: A Modular Approach to Machine Learning in Atomistic Simulations. Comput. Phys. Commun. 2016, 207, 310–324. 10.1016/j.cpc.2016.05.010.

[ref48] Li Z.; Kermode J. R.; De Vita A. Molecular Dynamics with On-the-Fly Machine Learning of Quantum-Mechanical Forces. Phys. Rev. Lett. 2015, 114, 096405 10.1103/PhysRevLett.114.096405. 25793835

[ref49] Keith J. A.; Vassilev-Galindo V.; Cheng B.; Chmiela S.; Gastegger M.; Müller K. R.; Tkatchenko A. Combining Machine Learning and Computational Chemistry for Predictive Insights Into Chemical Systems. Chem. Rev. 2021, 121, 9816–9872. 10.1021/acs.chemrev.1c00107. 34232033 PMC8391798

[ref50] Ko T. W.; Finkler J. A.; Goedecker S.; Behler J. A Fourth-Generation High-Dimensional Neural Network Potential with Accurate Electrostatics Including Non-Local Charge Transfer. Nat. Commun. 2021, 12, 398 10.1038/s41467-020-20427-2. 33452239 PMC7811002

[ref51] Yao K.; Herr J. E.; Toth D. W.; Mckintyre R.; Parkhill J. The TensorMol-0.1 Model Chemistry: A Neural Network Augmented with Long-Range Physics. Chem. Sci. 2018, 9, 2261–2269. 10.1039/C7SC04934J. 29719699 PMC5897848

[ref52] Grisafi A.; Ceriotti M. Incorporating Long-Range Physics in Atomic-Scale Machine Learning. J. Chem. Phys. 2019, 151, 204105 10.1063/1.5128375. 31779318

[ref53] Hoja J.; Medrano Sandonas L.; Ernst B. G.; Vazquez-Mayagoitia A.; DiStasio R. A. Jr.; Tkatchenko A. QM7-X, a Comprehensive Dataset of Quantum-Mechanical Properties Spanning the Chemical Space of Small Organic Molecules. Sci. Data 2021, 8, 43 10.1038/s41597-021-00812-2. 33531509 PMC7854709

[ref54] Blum L. C.; Reymond J.-L. 970 Million Druglike Small Molecules for Virtual Screening in the Chemical Universe Database GDB-13. J. Am. Chem. Soc. 2009, 131, 8732–8733. 10.1021/ja902302h. 19505099

[ref55] Ramakrishnan R.; Dral P. O.; Rupp M.; von Lilienfeld O. A. Quantum Chemistry Structures and Properties of 134 Kilo Molecules. Sci. Data 2014, 1 (1), 140022 10.1038/sdata.2014.22. 25977779 PMC4322582

[ref56] Ruddigkeit L.; van Deursen R.; Blum L. C.; Reymond J.-L. Enumeration of 166 Billion Organic Small Molecules in the Chemical Universe Database GDB-17. J. Chem. Inf. Model. 2012, 52, 2864–2875. 10.1021/ci300415d. 23088335

[ref57] Chmiela S.; Tkatchenko A.; Sauceda H. E.; Poltavsky I.; Schütt K. T.; Müller K. R. Machine Learning of Accurate Energy-Conserving Molecular Force Fields. Sci. Adv. 2017, 3, 1603015 10.1126/sciadv.1603015. PMC5419702 28508076

[ref58] Chmiela S.; Vassilev-Galindo V.; Unke O. T.; Kabylda A.; Sauceda H. E.; Tkatchenko A.; Müller K. R. Accurate Global Machine Learning Force Fields for Molecules with Hundreds of Atoms. Sci. Adv. 2023, 9, adf0873 10.1126/sciadv.adf0873. PMC9833674 36630510

[ref59] Smith J. S.; Zubatyuk R.; Nebgen B.; Lubbers N.; Barros K.; Roitberg A. E.; Isayev O.; Tretiak S. The ANI-1ccx and ANI-1x Data Sets, Coupled-Cluster and Density Functional Theory Properties for Molecules. Sci. Data 2020, 7, 134 10.1038/s41597-020-0473-z. 32358545 PMC7195467

[ref60] Fonseca G.; Poltavsky I.; Vassilev-Galindo V.; Tkatchenko A. Improving Molecular Force Fields across Configurational Space by Combining Supervised and Unsupervised Machine Learning. J. Chem. Phys. 2021, 154, 124102 10.1063/5.0035530. 33810678

[ref61] Fu X.; Wu Z.; Wang W.; Xie T.; Keten S.; Gomez-Bombarelli R.; Jaakkola T. Forces Are Not Enough: Benchmark and Critical Evaluation for Machine Learning Force Fields with Molecular Simulations. 2022, arXiv:2210.07237. arXiv preprint.

[ref62] Ewig C. S.; Berry R.; Dinur U.; Hill J.-R.; Hwang M.-J.; Li H.; Liang C.; Maple J.; Peng Z.; Stockfisch T. P.; Thacher T. S.; Yan L.; Ni X.; Hagler A. T. Derivation of Class II Force Fields. VIII. Derivation of a General Quantum Mechanical Force Field for Organic Compounds. J. Comput. Chem. 2001, 22, 1782–1800. 10.1002/jcc.1131. 12116411

